# Patient safety culture from the perspective of workers and primary health care teams

**DOI:** 10.11606/S1518-8787.2019053000788

**Published:** 2019-04-26

**Authors:** Daiane Cortêz Raimondi, Suelen Cristina Zandonadi Bernal, Laura Misue Matsuda

**Affiliations:** I Universidade Estadual de Maringá. Programa de Pós-Graduação em Enfermagem. Maringá, PR, Brasil

**Keywords:** Patient Safety, Primary Health Care, Manpower, Health Personnel, Health Knowledge, Attitudes, Practice, Segurança do Paciente, Atenção Primária à Saúde, recursos humanos, Pessoal de Saúde, Conhecimentos, Atitudes e Prática em Saúde

## Abstract

**OBJECTIVE:**

Analyze if the patient safety culture among professionals in the primary health care differs among health care teams.

**METHODS:**

Cross-sectional and quantitative study conducted in April and May 2017, in a city in Southern Brazil. A total of 144 professionals who responded to the questionnaire “Survey on Patient Safety Culture in Primary Health Care” participated in the study. Data were analyzed in the Statistical Analysis Software program and expressed in percentage of positive responses. The ethical principles established for research with human beings were applied.

**RESULTS:**

Patient safety culture is positive among 50.81% of the professionals, and the dimensions “your health service” (63.39%) and “patient safety and quality” (61.22%) obtained the highest average of positive responses. Significant differences were found between the family health and oral health teams (α = 0.05 and p < 0.05), in the dimensions “patient safety” (p = 0.0274) and “work at the health service” (p = 0.0058).

**CONCLUSIONS:**

We concluded that, although close to the average, patient safety culture among professionals in the Primary Health Care is positive and that there are differences in safety culture between family health and oral health teams in comparison with the primary health care teams.

## Abstract (pt)

**OBJETIVO:**

Analisar se a cultura de segurança do paciente entre os profissionais da atenção primária à saúde difere entre as equipes de saúde.

**MÉTODOS:**

Estudo transversal, quantitativo, realizado nos meses de abril e maio de 2017, em um município da região Sul do Brasil. Participaram 144 profissionais que responderam ao questionário “Pesquisa sobre Cultura de Segurança do Paciente para Atenção Primária”. Os dados foram analisados no programa *Statistical Analysis Software* e expressos em porcentagens de respostas positivas. Os preceitos éticos estabelecidos para pesquisas com seres humanos foram cumpridos.

**RESULTADOS:**

A cultura de segurança do paciente é positiva entre 50,81% dos profissionais e as dimensões “seu serviço de saúde” (63,39%) e “segurança do paciente e qualidade” (61,22%) obtiveram as maiores médias de respostas positivas. Identificou-se diferenças significativas entre as equipes de saúde da família e de saúde bucal (α = 0,05 e p < 0,05), nas dimensões “segurança do paciente” (p = 0,0274) e “trabalho no serviço de saúde” (p = 0,0058).

**CONCLUSÕES:**

Concluiu-se que, apesar de próxima da média, a cultura de segurança do paciente entre os profissionais da Atenção Primária à Saúde é positiva e existem diferenças na cultura de segurança entre as equipes de saúde da família e saúde bucal em relação às equipes de atenção básica.

## INTRODUCTION

Primary Health Care (PHC), main gateway of the Brazilian Unified Health System (SUS), is composed of teams that work in defined territory. The PHC teams have sanitary responsibility, i.e. they have to offer comprehensive care to individuals registered in their area of influence, developing health promotion, disease prevention, diagnosis, treatment, recovery and rehabilitation. They are also responsible for coordinating the care to the individual and the planning of the Health care Network^1^. This network, in turn, is characterized by organized health services aiming to promote a good, integral, efficient, safe and decisive health care to the population^1^.

More than 70% of the health care provided to the population are performed by the PHC teams^2^, as the health unit is located next to the residence of the patient, and it develops constant assistance at home and in the community. From this perspective, the PHC tends to offer decisive assistance, able to solve up to 85% of the health problems of the population^3^.

Even with the high demand for care and the resolution capacity of primary care, the health care provided by the PHC teams can result in errors or harm to patients^4^. A study conducted in Spain^4^ in 2007, whose aim was to investigate the patient safety in primary health care, found that 18.63% of the appointments made by medical professionals and nurses working at the PHC have caused some adverse events to the patients. Confirming these data, the survey conducted by the Health Foundation of London^5^ identified that the harm resulting from the care provided by PHC oscillate between 1% and 24%.

In Brazil, a study carried out between 2013 and 2014^6^ with family health teams (FHt) identified incidence rate of 1.11% in the health care provided in PHC. Among these incidence rates, 82% caused harm, from which 50% were of low or moderate severity, 25% were of high severity or with permanent harm, and 7% resulted in the death of the patient^6^. Another survey from 2009, conducted in primary health care centers, found that the errors in the PHC units are often related to miscommunication, lack of qualification and knowledge of professionals, inadequate exams and treatments and administrative service failures^7^.

Given this scenario, to ensure comprehensive, decisive and safe care in the PHC teams is paramount because patient safety, defined as the reduction of risk of unnecessary harm to an acceptable minimum related to health care^8^, is an essential dimension to the quality of health care. The patient safety culture, on the other hand, is the set of skills, values and behaviors that define the commitment to the health care safety and management^8^.

Aiming to prevent the errors in primary health care, the National Patient Safety Program (PNSP) was established in Brazil in 2013. Its purpose is to assist the institutions in the qualification of the health care provided in the country and promote patient safety culture for an effective care^9^. In the context of PHC, the National Policy of Primary Health Care (Ordinance 2,436, September 21, 2017) highlights the need for the implementation of the patient safety culture in its units to encourage safe and good practices and avoid errors and adverse events in the care process^1^.

Considering the relationship between patient safety culture and promotion of safe and good care, this study was based on the following questions: What is the existing patient safety culture among professionals of PHC? Is there any difference in the patient safety culture among PHC teams? To answer these questions, this study aimed to identify the patient safety culture among professionals of PHC and analyze if this culture differs among health teams.

## METHODS

Cross-sectional study with a quantitative approach, developed with PHC health teams from a city in Southern Brazil, including: FHt, primary health care team (PHt), and the oral health team (OHt).

Data were collected in April and May 2017 with professionals who met the inclusion criteria: being a nurse, nursing assistant or technician, physician, dentist, community health agent (CHA) and oral health assistant or technician; and work for at least 12 months at a unit that had the PHC teams mentioned. Exclusion criteria included: professionals on vacation, long paid leave and with medical certificate; pharmaceutical professionals, general services assistants, interns of the undergraduate course in pharmacy, administrative assistants and interns from high school or higher education who worked in the administrative service; and professionals who have not responded to the whole questionnaire. Pharmaceutical professionals were excluded from the survey because, at the time of data collection, the city investigated had only three pharmacists working at the PHC units.

The city in focus has six FHt, 22 PHt and 17 OHt, which totals 297 professionals. Of these, 144 professionals participated in this study, as 54 were excluded because they were on vacation, long paid leave or absent due to health problems, as well as the 18 participants who worked in the team for less than 12 months and the 59 people who did not accept to participate in the research. In addition, 22 people did not fully respond to the questionnaire and were also excluded.

Data collection was carried out with application of the instrument Medical Office Survey on Patient Safety Culture (MOSPSC), translated and adapted to Brazil, in 2016, with the title “Survey on Patient Safety Culture in Primary Care”^10^. This data collection instrument was chosen for being the only questionnaire about patient safety culture in PHC adapted to the Brazilian context. Its use was authorized by the authors responsible for the translation, cross-cultural adaptation and validation into the Brazilian context.

At the moment, this is the first study to apply the questionnaire “Survey on Patient Safety Culture in Primary Care”^10^ to workers and PHC teams. The instrument “Medical Office Survey on Patient Safety Culture” (MOSPSC) elaborated in 2007 was later adapted and validated into PHC in Spain and validated into Arabic and applied to Al Mukalla, in Yemen. In addition, the instrument was analyzed by experts of the United Kingdom, the Netherlands, Denmark, Germany, Poland and Austria, and the results showed that the questionnaire is a useful tool and applicable to analyze patient safety culture in primary health care in Europe^10^.

It is noteworthy that the instrument used for collecting data in this study allows us to verify if the patient safety culture in PHC is positive, when the average of positive responses is above 50%, and identify areas that need improvements. This instrument is composed of nine sections. From section A to G, the answers are arranged in Likert-type scales; section H has multiple-choice questions, and the Section I has only one open-ended question.

As to the instrument of data collection, about dimensions that compose the sections and the number of questions, we have^10^: section A – patient safety and quality: 10 questions; section B – exchange of information with other institutions: four questions; section C – working at the health service: 15 questions; section D – communication and follow-up: 12 questions; section E – support of public administrators/leaders: four questions; section F – your health service: seven questions; section G – global assessment: two questions; section H – professional practice: three questions; section I – your comments: a discursive question.

Upon the approval of the design of this study by the Ethics Committee, the Primary Health Care Coordination of the city was contacted for knowledge and authorization for the beginning of the research. Once the authorization from the Secretariat of Health was obtained, the nurses responsible for each team were contacted to define the beginning of data collection.

At the time of data collection, the researcher provided information regarding objectives, data collection and confidentiality. After that, the professionals were invited to participate in the survey, during the work shift, in the units where they work. Upon the verbal acceptance and signature of the informed consent form, the instrument of data collection was handed and then collected at the end of the work shift by the nurse of the team. In order to ensure the confidentiality of the participant, there was no identification in the questionnaire, which was deposited in a sealed envelope.

Data were tabulated in the Statistical Analysis Software program (SAS, version 9.4) from the database created in an electronic spreadsheet. Data processing and analysis were carried out in compliance with the recommendations of the Agency for Health Care Research and Quality (AHRQ), which adopts the percentage of positive responses^11^. The final score of each dimension was obtained by the average percentage of positive responses, including: “several times in 12 months,” “once or twice in 12 months” and “it did not happen”; “totally agree” and “I agree”; “almost always” and “always”; “good,” “very good” and “excellent.” In negative questions, the answers “totally disagree” and “disagree” were considered positive to the patient safety culture.

The dimensions of the patient safety culture were also classified according to the AHRQ guidelines: “strengths” when they show an average percentage of positive responses equal to or higher than 75%; and “weaknesses” when the average percentage is lower than 60%, which suggests the need for improvements in the dimension analyzed^11^.

To assess the difference among the total scores assigned to different dimensions, which compose the patient safety culture, among the PHC teams, the Kruskal-Wallis test was applied, followed by *post hoc* Dunn’s multiple comparisons test. In this process, the level of confidence of 95% and p < 0.05 were considered.

This study was developed according to the ethical principles established in Resolution 466/2012 of the National Commission of Ethics in Research, of the National Health Council^12^, and after the assent of the Standing Committee for Research Ethics on Living Creatures of the Universidade Estadual de Maringá, under Opinion 1,963,656 and CAAE 64095117.6.0000.0104

## RESULTS

A total of 144 professionals participated in the study, including: 16 nurses, eight doctors, 31 nursing assistants and technicians, 63 CHA, 15 dentists and 11 oral health assistants or technicians. As to the professional and sociodemographic profile, 132 (91.6%) were women; 82 (56.9%) were aged between 30 and 49 years; 94 (65.27%) were married; 78 (54.16%) people were working at the service for one to five years; and 66 (45.83%) were working at the service for six years or more.

Table 1 shows the frequencies and respective percentages to the positive responses of the dimensions analyzed. The overall average of positive responses of the dimensions that make up the safety culture was 50.81% among professionals who work in teams associated with PHC, indicating that, although very close to the average, the patient safety culture is positive. Among the dimensions, those that referred to “your health service” (63.39%) and “patient safety and quality” (61.22%) showed the highest averages for positive responses among the professionals who participated in this study. The dimensions “work at the health service” (48.52%), “support of public administrators” (40.80%) and “overall quality assessment” (32.64%) were negative.

**Table 1:** Positive responses of the dimensions of patient safety culture, according to professionals working at Primary Health Care in a city in Paraná. Brazil, 2017.

Dimensions	Positive responses	

	n	%
Patient safety and quality	884	61.2
Exchange of information with other institutions	305	53.0
Working at this service	1,048	48.5
Communication and follow-up	970	56.1
Support of public administrators	235	40.8
Your health service	639	63.4
Overall quality assessment	235	32.6
Overall average of positive responses	4,316	50.8

Table 2 shows the differences in the dimensions of patient safety culture established among the teams associated with PHC. These teams show percentage differences among the dimensions analyzed. In dimensions “patient safety” and “working at the health service,” there were significant differences between FHt and OHt in relation to PHt. However, in the dimensions “exchange of information,” “communication and follow-up,” “support of public administrators,” “health care service,” and “overall quality assessment,” FHt, OHt and PHt did not show significant differences.

**Table 2:** Differences of the dimensions of the patient safety culture among health teams working at the Primary Health Care in a city in Paraná. Brazil, 2017.

Dimension	FHt		PHt		OHt		p
		
	n	%	n	%	n	%	
Patient safety	611	57.6^a^	93	77.5^b^	180	69.2^a^	0.0274*
Exchange of information	211	49.7	35	72.9	59	56.7	0.1517
Working at this service	726	48.9^a^	120	71.4^b^	202	55.4^a^	0.0058*
Communication and follow-up	698	54.8	95	65.9	177	56.7	0.3548
Support of public administrators	179	42.2	20	41.5	36	34.6	0.4038
Health service	464	62.5	56	66.6	119	65.3	0.7717
Overall quality assessment	160	30.1	17	28.3	58	44.6	0.1849

* Significant at the 95% level (Kruskal-Wallis). Different letters indicate significant differences (p < 0.05) detected by the *post hoc* Dunn’s multiple comparisons test.

## DISCUSSION

The positive patient safety culture is present in PHC. In this study, positive responses were presented in four dimensions that make up the patient safety culture. Despite this, the culture still presents itself as a weakness because the overall average of positive response was lower than 60%, which indicates the need for improvements in the positive safety culture in PHC teams, ensuring thus a safe and good care to patients.

About the dimensions, a similar result was observed in a Brazilian research^13^ also developed with professionals from PHC, in which the “patient safety” showed the highest average of positive responses among professionals, being identified as the main dimension of patient safety culture.

The overall average of patient safety culture in the PHC of this study differs from others conducted in different countries such as Iran, developed in 2010^14^, which found positive patient safety culture in 57% of the PHC centers. In Al Mukalla, Yemen^15^, the percentage was 67%. The studies mentioned showed average of positive responses of the patient safety culture superior to this research, which reinforces the need for improvements in the work process of the PHC teams studied.

In the Netherlands, a study that also observed positive culture among professionals of the PHC teams^16^ highlights that the positive culture contributes to patient safety, because it promotes a safe and good care to the population. In Brazil^17^, the patient safety culture is indicated to help reduce errors and harms to health.

We observed that five dimensions analyzed showed a percentage of positive responses below 60%. These dimensions are the weaknesses of the patient safety culture in PHC teams.

It is noteworthy that the dimensions “communication and follow-up” and “exchange of information with other institutions” are positive, but they are considered weaknesses of the patient safety culture and require interventions to improve communication and the exchange of information, ensuring thus the safety of the patient. Considering the exchange of information related to communication^18^, failures in this process may be related to the lack of communication, communication or exchange of wrong and incomplete information, lack of integration and communication among professionals of the team or among other parts of the Health Care Network.

We reinforce that miscommunication and exchange of information between team, public administrators and Health Care Network can result in unsafety assistance, causing errors in patient care. In this regard, a systematic review^18^ aiming to identify incidents and the contributing factors to these occurrences in PHC, found that miscommunication is the main contributing factor to the occurrence of errors in PHC. Another study^7^ held in 2009, in primary health care centers, found that the errors in the PHC are generally related to miscommunication, professional inability, inadequate treatments and exams and failures in the administrative service.

Given the context, it is noteworthy that an effective communication contributes to a comprehensive and safe care, in addition to reducing the occurrence of errors^18^. To ensure safe assistance with communication and effective processes, authors^19^ suggest work valuation strategies, qualification with simulation techniques of cases with multidisciplinary teams, identification of failures and communication protocols to improve the process in the teams, administration, and health care network.

The dimensions “work at the health service,” “support of public administrators” and “overall quality assessment” showed the lowest percentages of positive patient safety culture among the professionals who work at the PHC. Therefore, there is a need for interventions in health teams to implement the positive patient safety culture.

Regarding the support of the public administrators, the professionals noted in the comments section the need for effective communication between public administrators and teams, correct allocation of resources and professional valuation. Thus, the involvement and good relationship of public administrators with professional teams are reflected on the care provided and on the safety of the institution^20^, reinforcing the idea that public administrators must support and empower workers to develop safe care and promote organizational safety culture.

The dimension “overall quality assessment” showed the lowest percentage of positive responses among professionals working at the PHC. Thus, there is indication that the professionals working at the PHC recognize faults in the processes of this dimension. This was also found in the study carried out in Al Mukalla^15^, in which only 47.5% of the professionals evaluated positively the patient safety and the quality of the service provided.

Professionals associated with the PHC in Turkey also show low percentage (42%) of positive response in the evaluation of the patient safety in the care provided in health units^21^. Studies point out^8^ ^,^ ^22^ that, to minimize the aforementioned situation, it is necessary to raise awareness of professionals for the commitment in identifying errors, teamwork, planning and implementation of changes, in order to manage the risks and implement the patient safety culture.

Regarding the comparison of the patient safety culture among the PHC teams, significant differences were found between the FHt and OHt in relation to PHt. This indicates that the patient safety culture among the teams associated with PHC needs improvements and actions to implement the positive culture in all the teams, so that safe and good assistance is guaranteed in all social assistance units.

The dimension “patient safety” is related to the circumstances that may occur in the PHC teams and compromise the safety and quality of the care provided, such as the access to health services, the errors in the identification of the patient, the use of medical records, medical prescription, exams and faults in the equipment^10^. Given this scenario, we can see that these situations occur most frequently with FHt and OHt than with PHt.

The dimension “work at this service” also showed differences among the teams studied, signaling that FHt and OHt present weaknesses and difficulties concerning teamwork; the valuation among professionals; failures in the process of permanent education, definition of workflows, and staff sizing. To minimize this problem, the literature^18^ suggests encouraging the exchange of information and knowledge among team workers, regular meetings to discuss the working process and clinical cases, with multidisciplinary activity; permanent trainings with professionals working at the PHC, and improvement in the administration of primary health care units.

It is noteworthy that in city studied, FHt and OHt act with adscript population greater than PHt, which tends to increase the demand for appointments and procedures, interfering with the differences introduced.

As limitations of this study, we include the need for a recall process to respond to the instrument used, the refusal of professionals to participate in the research, and the analysis of patient safety culture in the teams associated with PHC in only one city. According to these limitations, future studies using different methodological approaches are recommended, such as the case of mixed methods, with a qualitative approach.

## CONCLUSIONS

We observed that the patient safety culture among professionals working at the PHC is positive, but with a percentage very close to the average. In addition, the patient safety culture among the PHC teams studied differed significantly: PHt showed averages of positive responses higher than FHt and OHt. These data indicate the need for interventions to implement the positive culture in all teams, ensuring thus a safe and good care in all units.

Thus, we suggest adjustments in the dimensions that showed the lowest percentages of positive patient safety culture, as well as in the positive ones that showed weaknesses. We highlight the importance of establishing effective communication among professionals, public administrators and the health care network in order to discuss the work process and identify errors and solutions, in addition to encouraging patient safety attitudes through teamwork and professional integration. The data obtained in this study could provide reflections upon the work process of the PHC teams, as well as improvements in the positive patient safety culture. The study highlights the importance of the cross-sectional approach of the theme “patient safety” in the curriculum of courses in the health field in order to discuss the theme within vocational training and sensitize future professionals about safe and good care.

It is noteworthy that, to date, this is the first study carried out in Brazil focusing on patient safety culture in PHC using the questionnaire “Survey on Patient Safety Culture in Primary Care.”
